# Indices of Glucose Homeostasis in Cord Blood in Term and Preterm Newborns

**DOI:** 10.4274/jcrpe.2819

**Published:** 2016-09-01

**Authors:** Afzal Ahmad, M. S. Rukmini, Charu Yadav, Ashish Agarwal, Poornima A. Manjrekar, Anupama Hegde

**Affiliations:** 1 Manipal University Kasturba Medical College, Department of Biochemistry, Mangalore, India

**Keywords:** cord blood, cortisol, glucose, insulin, insulin resistance

## Abstract

**Objective:**

According to the thrifty phenotype hypothesis, intrauterine malnutrition has a role in the etiology of type 2 diabetes. This study was planned to determine the early alterations in indices of glucose homeostasis (glucose, insulin, and cortisol) in term and preterm newborns and the correlations of glucose, insulin, and cortisol levels with insulin resistance indices.

**Methods:**

A descriptive study comprising 35 term and 35 preterm newborns was carried out from December 2013 to June 2015. Venous cord blood was collected and plasma glucose was analyzed by the glucose oxidase-peroxidase method in an auto analyzer. Serum insulin and cortisol levels were assessed by the enzyme-linked immunosorbent assay. Homeostasis model assessment of insulin resistance (HOMA-IR), quantitative insulin sensitivity check index and glucose insulin ratio were calculated to assess insulin resistance. The data on physical and metabolic parameters were analyzed using standard tests for statistical significance.

**Results:**

In term newborns, mean glucose and cortisol levels (83.6±17.4 mg/dL and 11.88±5.78 µg/dL, respectively) were significantly higher than those in preterm infants (70.4±15.8 mg/dL and 8.9±4.6 µg/dL, respectively). Insulin and HOMA-IR levels were found higher in preterm newborns (10.8±4.8 µIU/mL and 1.52±0.66, respectively) than in term newborns (7.9±2.7 µIU/mL and 1.19±0.29, respectively). Insulin was found to positively correlate with HOMA-IR, whereas cortisol was negatively correlated with HOMA-IR in both term and preterm newborns.

**Conclusion:**

Higher insulin levels and HOMA-IR values in the cord blood of preterm newborns support the theory of intrauterine origin of metabolic diseases.

**WHAT IS ALREADY KNOWN ON THIS TOPIC?**

Thrifty phenotype hypothesis states that the etiology of type 2 diabetes mellitus occurs early during intrauterine development. In few studies, term newborns’ cord blood glucose, insulin, homeostasis model assessment of insulin resistance (HOMA-IR), and quantitative insulin sensitivity check index (QUICKI) were reported.

**WHAT THIS STUDY ADDS?**

As per our knowledge, for the first time in India, cord blood glucose, insulin, and cortisol levels in preterm and term newborns were estimated and insulin resistance was calculated using HOMA-IR, QUICKI, and glucose insulin ratio.

## INTRODUCTION

The newborn is considered to be in a transitional phase from a mother-dependent status to an independent-of-mother status. At delivery, the continuous transplacental flow of nutrients from the mother to the fetus stops abruptly. The successful transition from intrauterine to extrauterine life requires adaptation to several changes (1). Factors such as placental flow, maternal and hormonal secretion may determine and influence fetal growth (2). In this context, the hormones insulin and cortisol play an important role (3,4). Adaptation processes to extrauterine life are difficult to accomplish by the premature neonate. Thus, infants born prematurely are at higher risk for disturbed glucose homeostasis. They frequently develop hypoglycemia as a result of small substrate stores and immature enzyme systems (5). However, surprisingly, prevalence of hypoglycemia is estimated to vary between 40 and 80% among very preterm infants (6). This condition is much rarer in late preterm and term infants, highlighting the central role of immaturity in the pathophysiology of glucose homeostasis in preterm newborns (7).

Thrifty phenotype hypothesis regarding etiology of type 2 diabetes signifies that poor nutrition in fetal and early infant life is detrimental to the development and function of the β cells of the islets of Langerhans (8). The fetal insulin hypothesis also proposes a relationship between inherited insulin resistance and altered growth mediated by insulin (9). Early alterations in insulin and cortisol hormones influencing glucose homeostasis increase the risk of developing insulin resistance and obesity later in life (8). Thus, elevated insulin levels during perinatal life may predispose the infant to development of diabetes mellitus in future life (10).

The hormone concentration in the fetal circulation changes both developmentally and in response to nutritional stimuli. Near term, there is an increase and decrease in the concentrations of insulin and cortisol, respectively, signaling maturation of the fetus (11,12). An adrenocortical hormone, cortisol, is well-known as the stress-responsive hormone and its blood concentration is used as a stress marker. It modulates a large number of physiological actions involved in metabolic, inflammatory, cardiovascular, and behavioral processes. The molecular mechanisms and the physiological effects of cortisol have been extensively studied. However, the involvement of cord blood cortisol action in the etiology of diabetes and insulin resistance has not yet been clarified in term and preterm newborns. Recent mounting clinical evidence and animal studies have attracted growing interest in the role of cortisol action in obesity and insulin resistance (13).

Limited studies have been reported on glucose homeostasis indices, insulin resistance, and cortisol levels in cord blood of term and preterm newborns. Hence, this study was planned with an objective to determine the early alteration in indices of glucose homeostasis in cord blood of term and preterm newborns and the correlations of glucose, insulin, and cortisol with insulin resistance indices.

## METHODS

This cross-sectional study comprised 35 term and 35 preterm newborns who were born at the constituent medical college hospitals during the 1.5 years between December 2013 and June 2015. All infants were products of vaginal deliveries. The study was approved by the Institutional Ethics Committee and informed consent was obtained from the mothers. The study population comprised of offspring of residents of southern India mainly from in and around the city of Mangalore.

All selected term newborns were between 37 and 41 6/7 weeks of gestational age and their birthweight was between 2.5 and 4 kg. The preterm newborns were between 24 and 37 weeks of gestational age and their weights varied between 1.5 and 2.5 kg. Only term and preterm newborns with a 5th-minute Apgar score >9 were included in the study. Mothers suffering from any infectious disease or having obstetric complications such as gestational diabetes, hypertension, kidney disease, thyroid disease, PCOD were excluded.

Venous cord blood (VCB) was collected under aseptic conditions from the umbilical cords of all 70 newborns. After delivery, but prior to expulsion of placenta, 3 mL blood was drawn from the umbilical cord into a plain and a fluoride vacutainer. Plasma glucose was determined within 4 hours of collection. The serum was stored at -20 0C until further analysis for insulin and cortisol by ELISA. All data regarding mother and newborn were collected from the hospital files.

Plasma glucose estimation was done by the glucose oxidase-peroxidase method (Agappe diagnostic kits, Ernakulam, Kerala) using a Roche Hitachi P800 auto-analyser (Roche Diagnostics GmbH, Mannheim). The coefficient of variation (CV) for intra- and inter-batch for glucose was <4%. Insulin levels were assayed based on sandwich principle in ELx 800 by BIO TEK^®^ Instruments, Inc. using an insulin ELISA kit manufactured by DRG, a German company. The CV for intra‑ and inter‑batch insulin assay was <3%. Cortisol levels were assayed based on sandwich principle in ELx 800 by BIO TEK^®^ Instruments, Inc., using a cortisol ELISA kit manufactured by Cal biotech, a USA company. The CV for intra‑ and inter‑batch insulin assay was <6%. Insulin resistance indices were calculated by three metabolic parameters. Homeostatic model assessment of insulin resistance (HOMA-IR) was calculated using the equation: HOMA-IR=fasting insulin (μU/mL) x fasting glucose (mg/dL)/405 (14). Quantitative insulin sensitivity check index (QUICKI) and glucose insulin ratio (GIR) were calculated manually by calculation (15,16).

### Statistical Analysis

The data were analyzed using the IBM SPSS Statistics version 20 (SPSS, Chicago, IL, USA). The parametric data were presented as means ± standard deviation (SD) and nonparametric data as medians (first quartile, third quartile). The student’s (independent-samples) t-test and the Mann-Whitney U test were used to compare mean differences between the two groups for parametric and nonparametric data, respectively. Pearson’s correlation coefficient was used to determine the significant association between variables. A p-value less than 0.05 was considered statistically significant.

## RESULTS

In this cross-sectional study of 70 newborns, there were 31 males (44%) and 39 females (56%). Mothers included in this study were between 20 and 38 years of age with a mean age of 26.59 years.

Maternal age and neonatal anthropometrical data (mean ± SD, range) are shown in Table 1. Of the term newborns, 37% (13/35) were males and 67% (22/35) were females, whereas in the preterm newborns, 51% (18/35) were males and 49% (17/35) were females. Term newborns weighed more and had higher anthropometry values (p<0.001) than preterm newborns.

Pearson’s correlation values between cord blood glucose, insulin, and cortisol levels and insulin resistance indices in term and preterm newborns are shown in Tables 2 and 3. In the term newborns, plasma glucose was positively correlated with GIR (r=0.793, p<0.001) but negatively correlated with HOMA-IR (r=-0.481, p<0.01). Insulin was negatively correlated with QUICKI and GIR (r=-0.715, p<0.001 and r=-0.84, p<0.001, respectively) but positively with HOMA-IR (r=0.91, p<0.001). Cortisol was negatively correlated with HOMA-IR (r=-0.3, p<0.03) but was found to positively correlate with GIR (r=0.351, p<0.039).

In preterm newborns, plasma glucose showed positive correlations with GIR (r=0.47, p<0.004) and negative correlations with QUICKI (r=-0.39, p<0.02). Insulin levels showed a positive correlation with HOMA-IR (r=0.95, p<0.001) but a negative correlation with QUICKI and GIR (r=-0.406, p<0.01 and r=-0.72, p<0.001, respectively). Cortisol was found to positively correlate with glucose and GIR (r=0.376, p<0.02 and r=0.47, p<0.004, respectively) but negatively with insulin and HOMA-IR (r=-0.367, p<0.03 and r=-0.48, p<0.02, respectively).

## DISCUSSION

In this cross-sectional study, we estimated the levels of cord blood glucose, insulin, and cortisol in term and preterm newborns and the correlations between glucose homeostasis indices and insulin resistance markers. Glucose homeostasis indices in this study were derived from the estimated glucose, insulin, and cortisol levels. These levels were 36-116 mg/dL, 3-21 µIU/mL, and 3-26 µg/dL, respectively in the cord blood of the newborns included in the study. The glucose, insulin, and cortisol concentrations determined in our study population concur with previously reported levels which were 37-113 mg/dL, 3-21 μIU/mL, and 7-31.3 µg/dL, respectively for glucose, insulin, and cortisol (15,16,17).

In term newborns, mean glucose levels (83.6±17.4 mg/dL) were significantly higher than in preterm newborns (70.4±15.8 mg/dL), whereas insulin levels were found significantly lower in term newborns (7.9±2.7 µIU/mL) than in preterm newborns (10.8±4.8 µIU/mL). This confirms the role of insulin in glucose utilization indicating that glucose concentration decreases as the insulin level increases. In the current study, the higher insulin levels in preterm newborns than term newborns ratifies its higher requirement in preterm newborns for their growth and development (16).

Different values of cord blood cortisol level of term newborns have been reported. Gesteiro et al (15), Kırımi and Gül (17), and Sano et al (18) have reported cord blood values for term newborns as 4.4-10.4 µg/dL, 5.73-21.5 µg/dL, and 70-313 ng/mL, respectively (16). In this present study, mean cortisol level for preterm newborns was 8.9±4.66 µg/dL (4.4-24 µg/dL), a value lower than that for term newborns, i.e. 11.88±5.78 µg/dL (3-26 µg/dL). This may be attributed to the major regulatory action of cortisol in the final maturation of the fetus and in neonatal adaptation at birth. The fetal cortisol level remains low till 30 weeks of gestation and then progressively rises to reach 200 µg/mL near term (17,19). At the last stage of pregnancy, cortisol level increases in parallel to the development of fetus. However, the substantial direct effect of cortisol on birth weight is not yet established (17).

In preterm neonates, the adaptation process is very difficult to accomplish as they are at a higher risk of altered glucose homeostasis. They frequently develop hypoglycemia as a result of small substrate stores and immature enzyme systems (1). Insulin sensitivity is the ability of insulin to decrease plasma glucose levels by suppressing hepatic glucose formation and stimulating glucose utilization in skeletal muscle and adipose tissue, while insulin resistance is described as an impaired biological response to insulin (15).

The HOMA-IR, QUICKI, and GIR indices have rarely been tested in cord blood of newborns (20). In the present study, significantly increased insulin levels and HOMA-IR values were noted in the cord blood of preterm newborns as compared to term newborns. This shows that low birthweight newborns were at a higher risk of developing obesity and type 2 diabetes in later life considering the immature growth of β cells of pancreas in preterm newborns (21). In this study, no significant differences were found for QUICKI and GIR between term and preterm newborns despite the fact that preterm newborns had higher values. This may be due to the low prevalence of insulin resistance in neonates than older children (1).

In term newborns, glucose was found to negatively correlate with HOMA-IR but showed a positive correlation with GIR. This confirms the glucose utilization effect of insulin along with the added role of receptors activity in term newborns. Insulin had a strong positive correlation with HOMA-IR and a negative correlation with QUICKI and GIR. However, these associations were more significant in term than in preterm newborns. Bleicher et al (22) have shown significant correlations between cortisol and HOMA-IR in pediatric patients, indicating that cortisol contributes to insulin resistance. In contrast to Bleicher et al (22) we found significant negative correlation between cortisol and HOMA-IR and significant positive correlation between cortisol and GIR. In support of this finding, Adam et al (23) have also reported that cortisol has negative association with insulin secretion from the pancreas thus causing hyperglycemia and insulin resistance.

Our data shows a significantly higher correlation of cord blood cortisol with GIR in preterm newborns as compared to term newborns. It is known that low birth weight is partially responsible for hyperactivity of the hypothalamic–pituitary-adrenal axis which causes a state of functional hypercortisolism. Thus, increased cortisol levels and greater responsiveness of the hypothalamic–pituitary-adrenal axis may play an important role in the development of metabolic syndrome at both central and peripheral level in later life (16).

The obtained levels of cord blood glucose, insulin, and cortisol in normal term infants (83.6±17.4 mg/dL, 7.9±2.7 µIU/mL, and 11.88±5.78 µg/dL, respectively) and in preterm infants (70.4±15.8 mg/dL, 10.8±4.8 µIU/mL, and 8.9±4.66 µg/dL, respectively) can be used as our own reference for further studies as there are no studies on cord blood normal levels in term and preterm newborns in India. Increased cord blood insulin level and HOMA-IR in preterm infants show a risk for developing insulin resistance in preterm newborns.

The foremost limitation to our study is that it reports cross-sectional data, which prevented us from drawing causal relationships. Longitudinal larger scale studies are required to validate our findings and show any incremental prognostic information about chances of developing diabetes and insulin resistance in term and preterm newborns.

In conclusion, we hope that the cord blood levels of glucose, serum insulin, and cortisol as well as the HOMA-IR, QUICKI, and GIR reported in this study may help other researchers to create reference ranges in term and preterm newborns. Higher insulin levels and HOMA-IR values in preterm newborns at birth supports the hypothesis that states which can lead to obesity, hyperinsulinemia, and insulin resistance in later life can have an intrauterine origin. Elucidation of the underlying mechanisms may offer an opportunity to influence body composition of the preterm newborns and therefore their susceptibility to future risk of developing diabetes and insulin resistance. The ever increasing incidence of diabetes in populations can be assessed with a new perspective at birth itself and also as an initiative for an early intervention.

## Ethics

Ethics Committee Approval: Kasturba Medical College, Mangalore, India and 18/9/2013, Informed Consent: It was taken.

Peer-review: External and Internal peer-reviewed.

## Figures and Tables

**Table 1:**
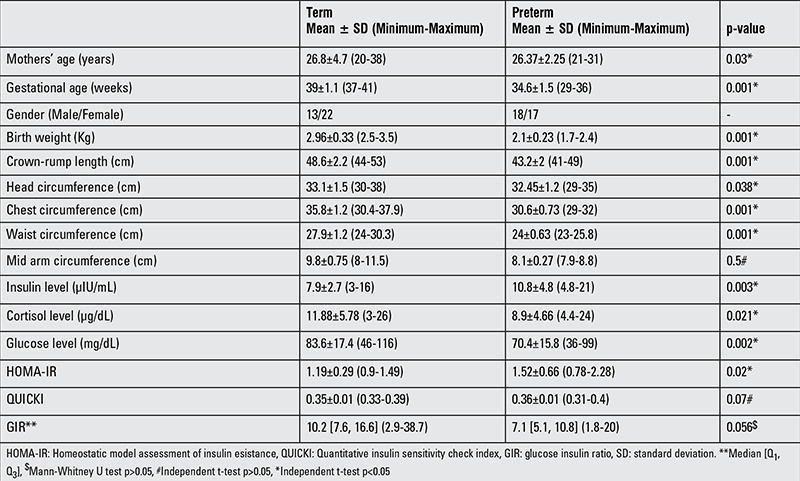
Descriptive data in term and preterm newborns and comparison of the two groups

**Table 2:**
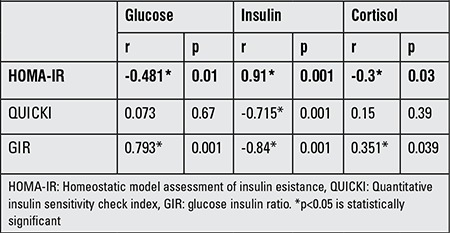
Correlations of cord blood glucose, insulin, and cortisol levels with insulin resistance indices in term newborns

**Table 3:**
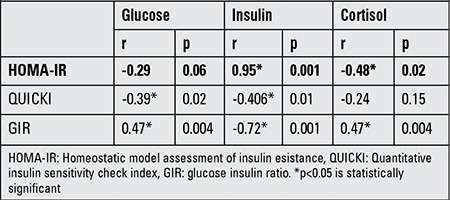
Correlations of cord blood glucose, insulin, and cortisol levels with insulin resistance indices in preterm newborns
